# Prevalence of Influenza A (H1N1) Seropositivity in Unvaccinated Healthcare Workers in Scotland at the Height of the Global Pandemic

**DOI:** 10.1155/2011/407505

**Published:** 2011-10-03

**Authors:** Kate Smith, Pamela Warner, Linda J. Williams, Walt E. Adamson, S. Vittal Katikireddi, Paul Dewart, William F. Carman, Kate Templeton, Fiona C. Denison, D. Graham Mackenzie

**Affiliations:** ^1^Public Health and Health Policy, NHS Lothian, Waverley Gate, 2-4 Waterloo Place, Edinburgh EH1 3EG, UK; ^2^Centre for Population Health Sciences, Medical School, University of Edinburgh, Teviot Place, Edinburgh EH8 9AG, UK; ^3^West of Scotland Specialist Virology Centre, Gartnavel General Hospital, Glasgow G12 0ZA, UK; ^4^St John's Hospital, Howden Road West, Livingston, West Lothian EH54 6PP, UK; ^5^Royal Infirmary Edinburgh, NHS Lothian, Edinburgh EH16 4SA, UK; ^6^MRC Centre for Reproductive Health, University of Edinburgh, Queen's Medical Research Institute, 47 Little France Crescent, Edinburgh EH16 4TJ, UK

## Abstract

*Background*. We set out to identify the level of previous exposure to influenza A (H1N1) in unvaccinated healthcare workers (HCWs) at the peak of the pandemic outbreak in the UK, with control samples collected prior to the outbreak. *Methods*. Cross-sectional study (seroprevalence assessed before and at pandemic peak, with questionnaire data collected at peak of outbreak) in HCWs in Scotland. *Results*. The prevalence of seropositivity in 493 HCWs at pandemic peak was 10.3%, which was higher than the prepandemic level by 3.7 percentage points (95% CI 0.3% to 7.3%, *P* = 0.048). Seropositivity rates for frontline and nonfrontline HCWs were similar. *Conclusion*. At pandemic peak, only 10.3% of HCWs were seropositive for influenza A (H1N1), so the great majority were still susceptible to infection at the introduction of the vaccination programme. Few studies have reported on seroprevalence in unvaccinated and asymptomatic participants, so our findings may have relevance to the wider population.

## 1. Introduction

The susceptibility of healthcare workers (HCW) to influenza is relevant in terms of sickness absence, productivity, and onward transmission of infection from carer to patient [1], a particular issue with a novel influenza strain. The first cases of 2009 pandemic influenza A (H1N1) were identified in Scotland in April 2009 [2]. We set out to determine the seroprevalence of antibodies against the virus in unvaccinated HCWs in Lothian, south east Scotland in Autumn 2009 (after the first wave of infection, but before the expected increase in cases over the winter period). It is now clear that this was close to the peak of the outbreak in the United Kingdom (UK) [3].

## 2. Materials and Methods

Between 29/10/2009 and 4/11/2009 (at the time believed to be midpandemic, but now known to have been peak), unvaccinated NHS Lothian employees (*n* = 505) were recruited within days of the start of the HCW vaccination programme and prior to most HCWs being vaccinated. Recruitment was mainly from three acute teaching hospitals, with smaller numbers from a psychiatric hospital and the Health Board headquarters. After giving informed consent participants had a serum sample taken and completed a short questionnaire recording sex, age, occupation, and self-reported history of flu-like symptoms or illness since the start of the pandemic period (April 2009). Of the 505 recruited, 493 employees were aged between 16 and 65 years and had complete information (laboratory results and questionnaire response). It is these 493 respondents who have been used in all analyses reported here. Serology specimens were tested for antibodies to influenza A (H1N1) in the West of Scotland Specialist Virology Centre, Glasgow, using microneutralisation assays at a dilution of 1 : 40 as previously described [4]. In addition a set of age- and sex-matched blood samples (*n* = 471) were obtained from stored serology specimens collected from HCWs for occupational health purposes during 2008 (i.e., prepandemic), and these were also tested. Chi-squared test with Yates' continuity correction was used to compare seropositive prevalence in pre- and peak pandemic samples and logistic regression analysis to examine the association of seropositivity with risk group classification. The study was approved by the Local Regional Ethics Committee. Sample size needed was calculated as 500, to ensure a pandemic estimate of seropositivity with 95% confidence interval no wider than ±3.5 percentage points, assuming that the observed prevalence was 20%.

## 3. Results

The age and sex profile for pre- and peak pandemic samples was similar to the overall hospital-based NHS Lothian workforce, and 67% of pandemic participants were classified as frontline, the same as the overall HCW workforce.

In the pandemic sample the prevalence of seropositivity in HCWs was 10.3% (95% confidence interval (CI) 7.7 to 13.0%). This seropositivity was higher than prepandemic HCW seropositivity rate by 3.7 percentage points (95% CI 0.3 to 7.3 percentage points, *P* = 0.048). The study was not powered to test for variation in seropositivity rates across age bands, but the highest pandemic rate observed was 17.4% in the youngest age band (16 to 25 years old), whereas prepandemic this age band had nearly the lowest rate (4.3%). However, the small numbers in the youngest age band means that the difference in seropositivity (pre versus pandemic) was not statistically significantly different according to whether comparing within those aged up to 25 years or over 25 years (*P* = 0.147). 

Occupations were grouped into frontline contact (allied health professionals, doctors, nurses, midwives, and students) and non-frontline contact (administrative, pharmacy, and support staff including e.g., individuals working in laboratories or “estates”). For pandemic samples, seropositivity rates for frontline and non-frontline HCWs were similar overall at 11.0% (95% CI 7.6 to 14.4%) and 9.1% (5.8 to 12.5%), respectively. Influenza-like symptoms in the preceding six months were reported by 208 (42.2%), and 12.0% (95% CI 7.6 to 16.4%) of them were seropositive, compared to 9.1% (95% CI 5.8 to 12.5%) of those without recent symptoms (Table 1).

**Figure 1:**
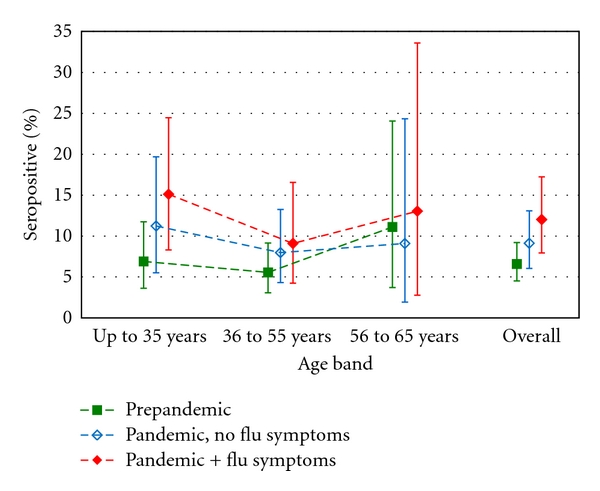
Seropositivity separately for peakpandemic and prepandemic samples, with peakpandemic samples subdivided according to report or not of influenza-like symptoms (*n* = 208, 285, 471, resp.), overall and by age band.

A trend analysis across three levels of risk (prepandemic, pandemic but no influenza-like symptoms, pandemic reporting symptoms) found a statistically significant trend in seropositivity (*P* = 0.018), with a linear odds ratio of 1.39 (95% CI 1.06 to 1.84), suggesting that overall the odds of a positive laboratory result increased by 39% for each move from one risk category to the next higher category.  Figure 1 shows overall seropositivity for the three risk categories and across age bands.

## 4. Discussion

To our knowledge, this is the first study in the UK to quantify the level of seropositivity to influenza A (H1N1) in unvaccinated HCWs pre- and peak pandemic. Our findings have important implications both for understanding the spread of influenza A (H1N1) and for planning and delivery of future pandemic influenza vaccination programmes. 

Previous vaccination is indistinguishable from previous infection on microneutralisation testing, so our active recruitment of unvaccinated participants for the pandemic samples avoids the limitation of other studies which did not have full documentation of vaccination status, many of which relied on discarded laboratory samples, samples from blood donors or patients [4–7]. Other studies have looked at a single time point [4], including one study of HCWs (from Taiwan) [8], which means that it is not possible to compare prepandemic immunity observed in other studies [6].

Our study included pre- and pandemic samples which allowed us to compare peak pandemic seropositivity against the rate about 9 months before the start of the pandemic. The only other HCW study conducted pre- and midpandemic that we have identified (from Singapore) used a different assessment of seroconversion, requiring a 4-fold rise in titre from baseline, so a direct comparison with our findings is not possible [9]. 

Elder has reported previously that there is little indication of increased susceptibility to seasonal influenza by occupational group, including healthcare [1]. Accordingly, in the absence of a more general study of seroprevalence in unvaccinated adults of working age, our estimate of pandemic seropositivity is arguably the closest estimate to date of overall population prevalence in adults in the UK at that time. 

It is important to remember the levels of uncertainty and concern that existed midpandemic. In October 2009 the Scottish Chief Medical Officer noted a slower spread than anticipated [10]. However that same week the United States declared an influenza A (H1N1) emergency, with 1,000 deaths across 46 states and questions about the ability to cope with a potential surge in cases [11]. A week later Health Protection Scotland reported that influenza A (H1N1) related hospitalisations and deaths in Scotland continued to increase [12]. Furthermore, the influenza virus's ability to mutate and transfer genetic material between strains meant that a substantial increase in cases over the winter influenza season remained a possibility. Our findings show that over the first six months of the pandemic the A (H1N1) strain was of limited virulence with only around 4% of susceptible HCWs having developed seropositivity despite 42% participants reporting flu-like symptoms in preceding months. However, previous virulence is not necessarily a guide to future spread, particularly on the cusp of the influenza season, and our findings show that 90% HCWs were still susceptible to infection. These findings support the importance of vaccination even at the peak of a pandemic (and regardless of previous symptoms), contrasting with findings of a large English study that sampled patients accessing health care between August and September 2009 [6]. 

It has been estimated that between 40 and 50% of HCWs in the UK were vaccinated for influenza A (H1N1) by February–April 2010 [13–15]; in Lothian the figure was 52% [16]. This high level of uptake in HCWs across the United Kingdom in the weeks following this study means that it would not be feasible to estimate postpandemic seropositivity among unvaccinated HCWs. Although in other studies performed towards the end of the pandemic period the seroprevalence reported—between 20 and 40% [4, 5, 7]—was considerably higher than we found, there is uncertainty about vaccination rates and caveats about study population in these studies, as described above. 

Our study has a number of potential weaknesses. We did not recruit primary care staff. Self-selection of participants and the exclusion of HCWs who were targeted first by the vaccination programme may have led to an under- or overestimation of the true level of infection with the virus for all HCWs in Lothian. Other studies have demonstrated a greater increase in seropositivity during the pandemic period in younger adults (16 to 25 years old) compared to older adults, both in high prevalence areas in the UK [4] and among blood donors in Australia [5]. While our study showed a similar pattern of greater increase in seropositivity in younger adults than other age groups (Figure 1) it was not powered to test such an interaction hypothesis. 

These findings have important implications for research into future pandemics. Having information on seroprevalence in unvaccinated individuals *during* the pandemic would have been invaluable and may be feasible in a future pandemic. Virology samples can be stored indefinitely allowing comparison with samples taken from previous years. Such information would help identify susceptible age groups, helping the planning of vaccination campaigns during the pandemic. We therefore suggest that health protection organisations consider collecting samples annually from a representative “panel” of asymptomatic individuals, selected and powered to allow comparisons by age.

## 5. Conclusion

In conclusion, our study shows that at pandemic peak 10.3% of HCWs in Lothian, south east Scotland, were seropositive for influenza A (H1N1), so the great majority were still susceptible to influenza A (H1N1) infection at the introduction of the vaccination programme.

## Figures and Tables

**Table 1:** Seropositivity overall and by age band, separately for prepandemic and peak pandemic samples.

Age band (years)	Prepandemic samples				Peakpandemic samples										
					Overall				Subdivided according to report of influenza-like symptoms						
									Reporting symptoms			No symptoms reported			
	Subgroup *n *	Seropositive *n *	% Seropositive		Subgroup *n *	Seropositive *n *	% Seropositive		Subgroup *n *	Seropositive *n *	% Seropositive	Subgroup *n *	Seropositive *n *	% Seropositive	

16–25	46	2	**4.3**		46	8	**17.4**		21	3	**14.3**	25	5	**20.0**	
26–35	128	10	**7.8**		129	15	**11.6**		65	10	**15.4**	64	5	**7.8**	
36–45	138	10	**7.2**		137	14	**10.2**		52	7	**13.5**	85	7	**8.2**	
46–55	114	4	**3.5**		125	8	**6.4**		47	2	**4.3**	78	6	**7.7**	
56–65	45	5	**11.1**		56	6	**10.7**		23	3	**13.0**	33	3	**9.1**	

Overall	471	31	**6.6**		493	51	**10.3**		208	25	**12.0**	285	26	**9.1**	

95% CI			**4.3 to 8.8**				**7.7 to 13.0**				**7.6 to 16.4**			**5.8 to 12.5**	
